# Efficacy and safety of a novel personalized navigation template in proximal femoral corrective osteotomy for the treatment of DDH

**DOI:** 10.1186/s13018-020-01843-y

**Published:** 2020-08-12

**Authors:** Qiang Shi, Deyi Sun

**Affiliations:** 1 Department of Sports Medicine, Xiangya Hospital, Central South University, Changsha, 410008 People’s Republic of China; 2 Key Laboratory of Organ Injury, Aging and Regenerative Medicine of Hunan Province, Changsha, 410008 People’s Republic of China

**Keywords:** Femoral corrective osteotomy, 3D printing, Navigation template, Developmental dysplasia of the hip

## Abstract

**Background:**

This present study is aimed to retrospectively evaluate the efficacy and safety of a novel personalized navigation template in proximal femoral corrective osteotomy for the treatment of DDH.

**Methods:**

Twenty-nine consecutive patients with DDH who underwent proximal femoral corrective osteotomy were evaluated between August 2013 and June 2017. Based on the different surgical methods, they were divided into the conventional group (*n* = 14) and navigation template group (*n* = 15). The osteotomy degrees, radiation exposure, and operation time were compared between the two groups.

**Results:**

No major complications relating to osteotomy surgery such as redislocation or avascular necrosis occurred in the navigation template group, which had more accurate osteotomy degrees, less radiation exposure, and shorter operation time when compared with the conventional group (*P* < 0.05). Moreover, there was significant difference according to the McKay criteria between the two groups (*P* = 0.0362).

**Conclusions:**

The novel personalized navigation template in proximal femoral corrective osteotomy is effective and safe, which could improve the femoral osteotomy accuracy, reduce radiation exposure, and shorten operation time.

## Background

Developmental dysplasia of the hip (DDH) is considered one of the most common three-dimensional (3D) hip deformities in children [1]. Derotational femoral shortening osteotomy as part of the surgical procedure of DDH was firstly introduced by Klisic and Jancovic as an aid to decrease the complications related with surgery such as redislocation or avascular necrosis [2]. Other authors have also regarded proximal femoral corrective osteotomy as an effective procedure for treating DDH [3–5]. The performance and positioning of femoral osteotomy is one of the most important surgical procedures to ensure ideal correction effects, but the conventional radiographic imaging methods, such as x-ray or CT (computed tomography), were not sufficient to provide accurate information about complex deformities [6]. Moreover, individual differences in the size and shape of femur remain changeable factors that may essentially influence the accuracy of femoral osteotomy.

Zheng et al. [7] designed a 3D-printed navigation template for the proximal femoral varus rotation and shortening osteotomy. However, it was doubtful using the template to correct angles of femoral anteversion and neck-shaft simultaneously due to different planes. Based on these concerns, the present study introduces a novel design and application of derotational femoral shortening osteotomy with navigation template for the treatment of DDH in children. Therefore, this study was performed to assess the efficacy and safety of navigation template for more accurate osteotomy degrees, less radiation exposure, and shorter operation time when compared with the conventional operation, thus to result in a clinically viable alternative which is simple and precise.

## Materials and methods

### Patients

From August 2013 to June 2017, 29 consecutive patients (7 males and 22 females) presenting DDH who underwent derotational femoral shortening osteotomy were included in our hospital. Patients with subluxation of hip or classified as grade I were not involved in this study. Based on different surgical methods, they were divided into the conventional group (*n* = 14) and navigation template group (*n* = 15). There was no significant difference between the two groups in terms of age, gender, side, Tönnis classification, and surgery history (Table 1). This study was approved by the Ethics Review Committee of Xiangya Hospital Central South University, and all parents in the present study signed an informed consent to participate.

**Table 1:** Comparison of demographic data and deformity characteristics between two groups

Characteristics	Conventional group (*n* = 14)	Navigation template group (*n* = 15)	*P* value
Mean age (range), years	4.2 ± 1.6 (3-8)	3.7 ± 2.2 (2–8)	0.478
Gender, *n* (%)			0.742
Male	3 (21.4)	4 (26.7)	
Female	11 (78.6)	11 (73.3)	
Side, *n* (%)			0.597
Left	8 (57.1)	10 (66.7)	
Right	6 (42.9)	5 (33.3)	
Tönnis classification			0.657
II	4 (57.1)	3 (20.0)	
III	7 (54.5)	10 (66.7)	
IV	3 (18.2)	2 (13.3)	
Surgery history			0.573
Yes	8 (57.1)	7 (46.7)	
No	6 (42.9)	8 (53.3)	

### Digital design and navigation template preparation

**Fig. 1:**
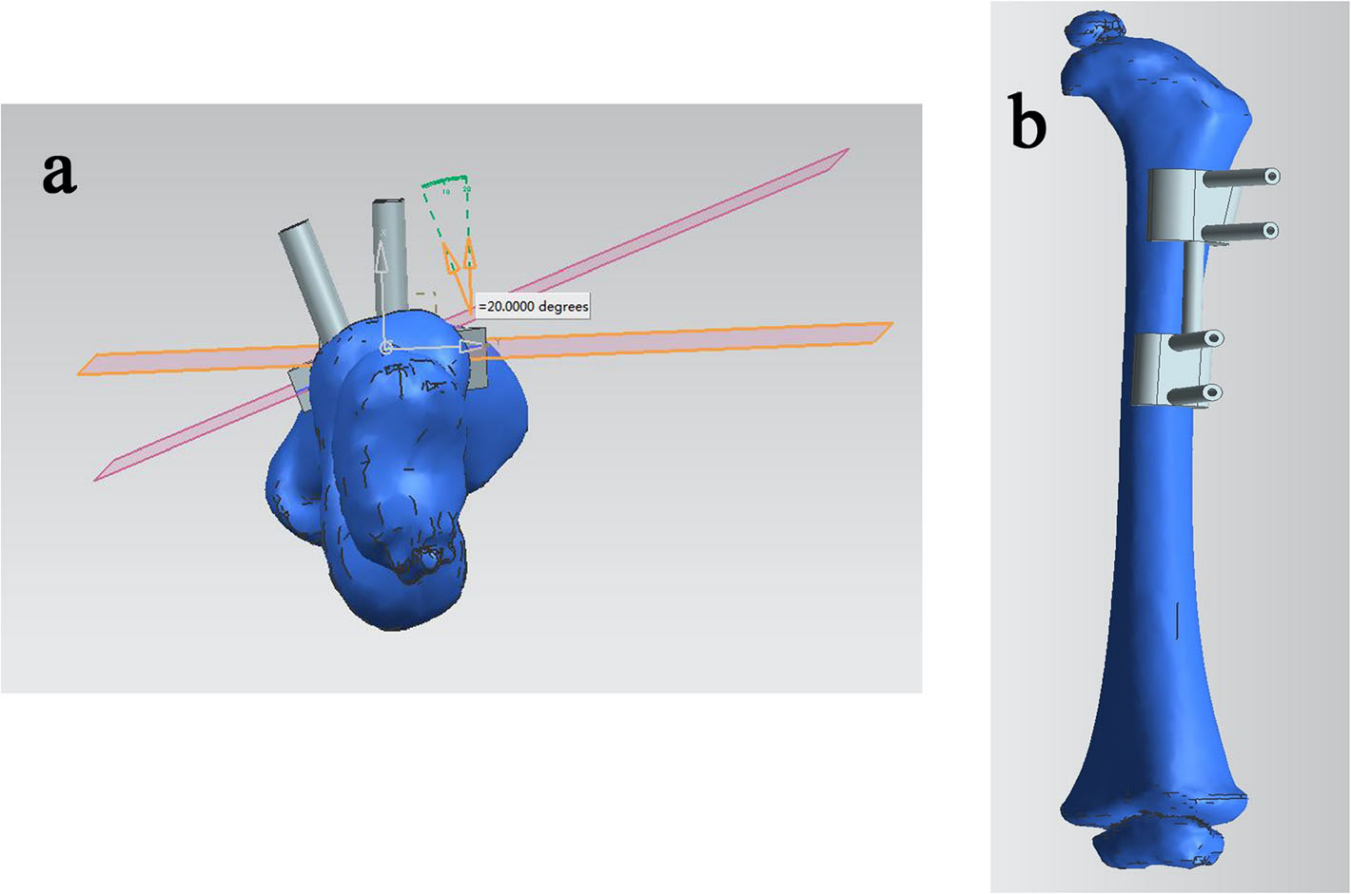
Design of osteotomy angle and plane. **a** The osteotomy plane and accurate planning for the degree of derotation were defined based on the femoral parameters. **b** Simulation of the navigational template on the femur

Preoperatively, all patients underwent x-rays and femoral CT scanning. The obtained slice CT scanning data in navigation template group were saved as DICOM (Digital Imaging and Communications in Medicine) format and then transferred into the Mimics 20.0 software (Materialise, Leuven, Belgium) for three-dimensional reconstruction. 3D models of femur were accurately reconstructed and exported in STL (Standard Tessellation Language) format. Thereafter, the deformed 3D models were imported into the UG 6.0 software. The osteotomy plane and accurate planning for the derotational degrees were defined based on the femoral parameters (Fig. 1a). And then, the personalized corrective osteotomy template model with four guide holes was fitted to the bone surface with reference to the characteristic configuration of the deformed femur and anatomical landmarks (Fig. 1b). The designed corrective osteotomy template was then printed using 3D printing technology with materials of acrylic resin.

3D reconstruction was performed using the Amira 3.1 software. By tracking the contours of the prominent bone structure for reconstruction, adjusting the geometric alignment of the overlapping point contours, modeling the surface by meshing into a polygonal point frame, and reconstructing the target contour based on the point data. The result is a fully interactive 3D visualization of the reconstructed structure based on radiology and CT imaging data, allowing full visualization of the area. The 3D models were saved in STL format for stereolithography, which is a universal international file standard for 3D modeling and printing, and then imported into the Imageware (version 12.0; EDS, Palo Alto, CA) software for further analysis. The optimal osteotomy plane and accurate planning for the derotational degrees were defined based on the femoral parameters using 3D printing technology. System parameters included the thickness of the processing layer at 0.1 mm, processing speed at 500 mm/s. The entire process of prototype construction required approximately 5–18 h, with an average of 8.9 h.

### Surgical procedures

**Fig. 2:**
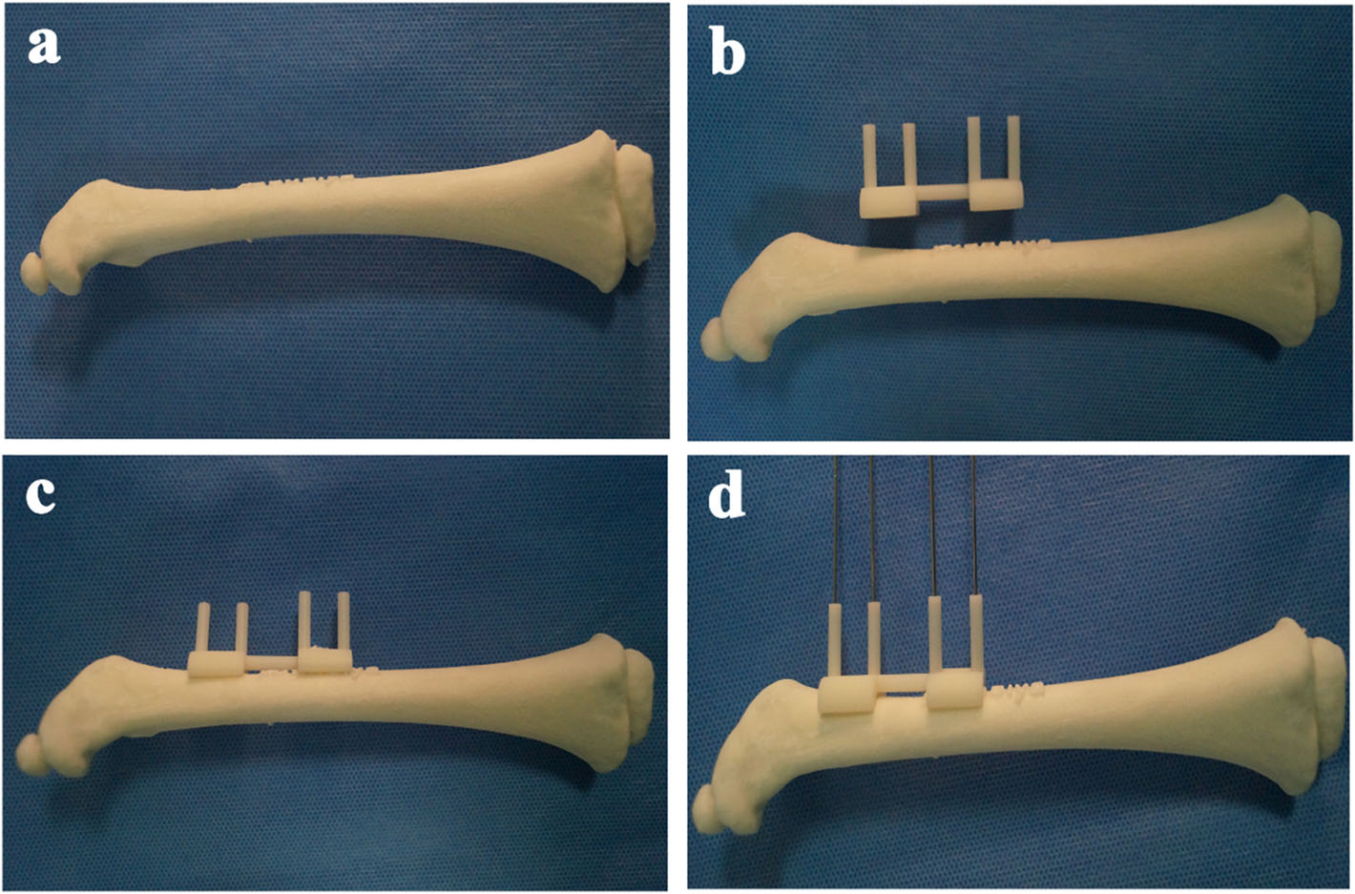
Simulated operation with 3D printing model and navigation template. **a** 3D printing model of the femur. **b** 3D printing model of the femur and navigational template. **c** The navigational template fitted 3D printing model of femur perfectly. **d** The K-wires was inserted through the navigational template

**Fig. 3:**
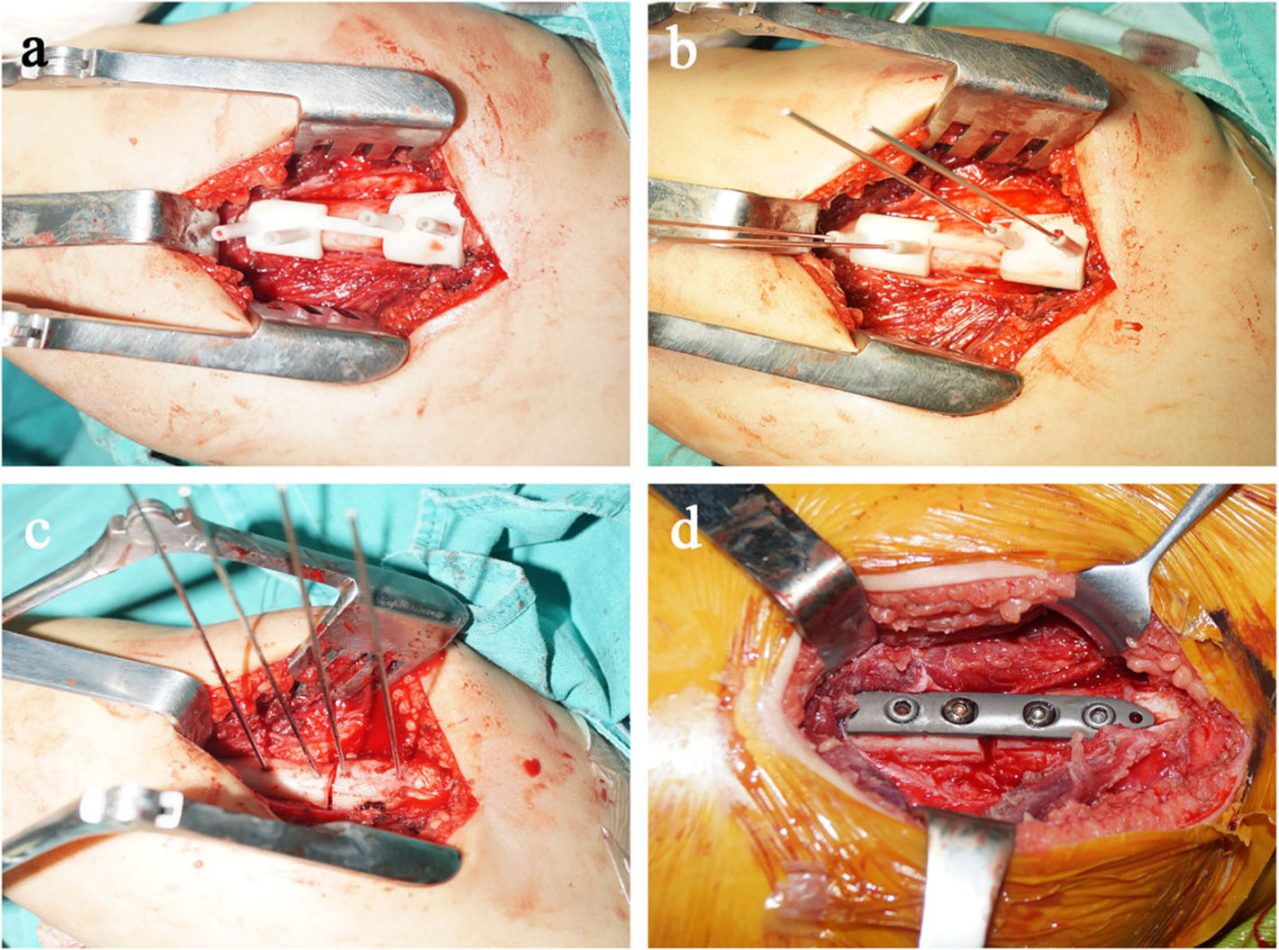
Intraoperative operation using the navigation template. a The navigation template fitted the surface of the femoral bone perfectly. **b** Four K-wires were inserted applying the template. **c** The template was removed, and a reduction guide was used to maintain the K-wire in a parallel position. **d** Internal fixation was then accomplished with LCP

All the proximal femoral corrective osteotomies in the present study were performed by one senior orthopedic surgeon in our department. The navigation template was sterilized and applied intraoperatively to assist derotational femoral shortening osteotomy based on preoperative simulation (Fig. 2). When the proximal femur was exposed, usually via a separate lateral incision, the navigation template was matched to the proximal lateral femur in the best possible manner (Fig. 3a). Four K-wires were inserted through the navigation holes of the template (Fig. 3b), then the bone was osteotomized through a slit on the template. The template was then removed with the K-wires left in place, matching with the shortening osteotomy bone which was required to provide a pressure-free reduction. Rotating the K-wires into the parallel status and this position was maintained with a reduction guide (Fig. 3c). Therefore, we were able to perform all osteotomies as preoperatively simulated. Finally, they were managed by internal fixation with Locking Compression Plate (LCP) (Fig. 3d). On the other hand, the derotational degrees of the osteotomy and shortening distance were performed on the basis of preoperative and intraoperative measurements in the conventional group.

### Postoperative management

No significant difference in postoperative management procedures after derotational femoral shortening osteotomy between the two groups. A spica cast fixation was used for 8 weeks and double lower limb brace with hip abduction for another 8 weeks. Weight bearing was guided to start after removal of the brace. Moreover, radiographs of the pelvis and femur were taken regularly until the region of osteotomy completely healed, and then the internal fixation was removed. The mean follow-up was 4.8 years (3 to 7 years), and patients were reviewed at 8 weeks, 4 and 12 months, and then every year until skeletal maturity.

### Statistical analysis

Quantitative data in this study were statistically analyzed by the SPSS 25.0 software (SPSS, Inc., Chicago, USA) and manifested as count (percentage) or mean ± standard deviation (SD). Student’s *t* test, chi-squared test, and Fisher’s exact test were applied to analyze the data in this study. Different parameters measured between two groups were evaluated with independent *t* test for continuous variables and chi-square test or Fisher’s exact test for the categorical variables. A *P* value < 0.05 was considered statistically significant.

## Results

There was statistical significance in osteotomy degrees (*P* = 0.0003), radiation exposure (*P* < 0.0001), and operation time (*P* < 0.0001) when compared with the conventional group (Table 2). Regarding the osteotomy degrees, there was statistical significance between the conventional group (23.6 ± 7.3°) and navigation template group (18.3 ± 5.4°, *P* = 0.0003). However, as for the shortening length, there was no statistical significance between the conventional group (2.3 ± 0.7 cm) and navigation template group (2.4 ± 0.3 cm, *P* = 0.1149). The operation time in the navigation template group (20.6 ± 3.9 min) was significantly less than that of the conventional group (37.3 ± 8.8 min, *P* < 0.0001). Based on the McKay criteria, 13 patients in the navigation template group obtained excellent correction, 1 patient obtained good prognosis, and 1 patient obtained fair prognosis, while 5 patients were scored as excellent, 5 as good, 1 as fair, and 1 as poor in the conventional group. As a consequence, the surgical outcome between the two groups was no significant difference (*P* = 0.0362).

**Table 2:** Comparison of operation data and functional outcomes between two groups

	Conventional group (*n* = 14)	Navigation template group (*n* = 15)	*P* value
Osteotomy degrees, °	23.6 ± 7.3	18.3 ± 5.4	0.0003
Shortening length, cm	2.3 ± 0.7	2.4 ± 0.3	0.1149
Radiation exposure, times	8.1 ± 2.8	3.0 ± 1.4	< 0.0001
Operation time, min	37.3 ± 8.8	20.6 ± 3.9	< 0.0001
McKay standard, *n* (%)			0.0362
Excellent	5 (35.7)	13 (86.7)	
Good	5 (35.7)	1 (6.7)	
Fair	2 (14.3)	1 (6.7)	
Poor	2 (14.3)	0	

Clinical assessment including measurement of leg-length inequality, hip range of motion, and gait pattern showed normal motion and gait. No patient had significant limb shortening, and no major complications relating to osteotomy surgery such as redislocation or avascular necrosis occurred in the navigation template group after 18 months of follow-up.

### Typical case

**Fig. 4:**
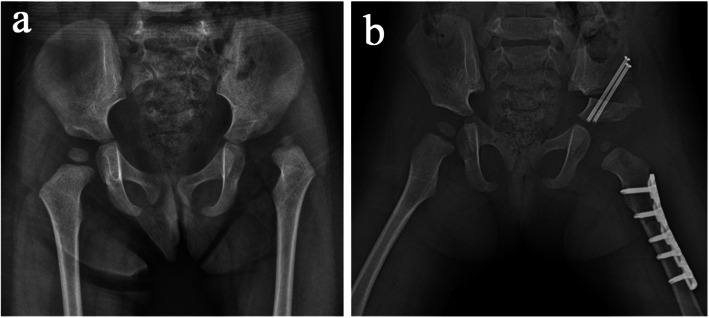
Radiographs before and after the operation. **a** Preoperative anteroposterior radiograph of left DDH in the navigation template group. **b** Postoperative radiograph showed anatomical correction and good appearance 1 week after surgery

A 2-year-old girl who was found limping by her parents came to our department, and radiographs of the pelvis showed that her left femoral head was dislocated from the normal position (Fig. 4a). According to the Tönnis classification, this girl was classified as grade III. Assisted by 3D navigation template, derotational femoral shortening osteotomy was performed, and the proximal femur was fixed at 20° of derotation with LCP (Fig. 4b). The bone healing of the femoral osteotomy was achieved 4 months after surgery. After 18 months of follow-up, normal motion and gait with leg-length equality were found, and the anteversion angle of the left femur was 17°, which was evaluated as excellent according to the McKay criteria [8].

## Discussion

Accurate operative procedures of DDH are technically challenging for surgeons. It is well accepted that derotational femoral shortening osteotomy is always necessary to facilitate the open reduction of DDH, especially in high dislocations and older children, correct abnormal femoral anteversion angle, reduce excessive pressure on the femoral head, and decrease the intraoperative instability of the hip [9]. In the guide-pins method, one pin was inserted into proximal and the other distal to the osteotomy position [10, 11]. When the distal pin lies parallel to the proximal pin, the desired derotation of the femur was achieved. The rate for overcorrection of affected legs was 44% and 15% for undercorrection, respectively. In the present study, we achieved satisfactory results in 15 cases by using 3D navigational template.

In order to complete the operation safely and achieve the desired effect of DDH, it is crucial to acquire as much deformity information in various situations as possible. Radiography, 2D, or 3D CT images alone cannot provide sufficiently accurate information about complex hip deformities [12, 13]. Size and shape differences in the proximal femur also affect the accuracy of osteotomy. Furthermore, due to limited exposure of lateral cortex of the proximal femur, performing derotational femoral shortening osteotomy accurately remains challenging [14]. Computer-aided navigation is an effective and useful tool to improve the accuracy of operation [15, 16]. However, they are expensive equipment, time-consuming procedures, and have a significant learning curve.

Recently, many 3D navigation templates have been introduced in the trauma, joint, and spine fields [17–19], but only a few researchers have reported their applications in the pediatric orthopedic disorders. Previously, different personalized templates have been designed for cubitus varus deformity and achieved wonderful clinical outcome [20–22]. Lu et al. [23] developed a novel patient-specific template in congenital scoliosis and validated the accuracy and safety. More recently, Otsuki et al. [24] constructed 3D custom cutting guide for curved peri-acetabular osteotomy. With its accurate guide, this template was considered to avoid serious complications associated with osteotomy. Compared with other templates, the present study had applied a novel personalized navigation template in proximal femoral corrective osteotomy for the treatment of DDH and validated the efficacy and safety in clinical settings. Neither redislocation nor avascular necrosis was detected in the navigation template group, which had more accurate osteotomy degrees, less radiation exposure, and shorter operation time when compared with the conventional group (*P* < 0.05). Moreover, there was significant difference according to the McKay criteria between the two groups (*P* = 0.0362).

The advantage of the 3D navigation template is that it is simple and easy to apply which can be performed without much expertise and special training. Moreover, the template can be used during surgery to assist surgical navigation and accurate osteotomy. Lastly, we can check the accuracy of individual template with the aid of 3D printing model before surgery, thereby eliminating the need for repeated manipulates and shortening the surgical time. Besides, fewer radiographs are needed, and continual fluoroscopic monitoring can be avoided intraoperatively.

Nevertheless, because the 3D navigation template is developed based on a preliminary series, it has several limitations in the present study. On the one hand, muscle and fat tissue on the bone can affect the position of the template. So, an exact preparation of the bone surface was essential, and the template should fit tightly to the corresponding lateral femur to allow accurate drill trajectories. Furthermore, more cases and multi-center prospective studies in the future are needed to evaluate the accuracy and efficacy of the 3D navigation template. In addition, it is necessary to further prolong the follow-up time because the patients in our study were still at the stage of skeletal immaturity.

## Conclusion

The application of a novel personalized navigation template for derotational femoral shortening osteotomy can simplify the operation and generate a highly accurate and reliable correction. Compared with the traditional freehand technique, which requires a long learning curve and experienced surgeons, the use of our 3D navigation template has the advantages of more accurate osteotomy degrees, less radiation exposure, and shorter operation time.

## Data Availability

The data used to support the findings of this study are available from the corresponding author upon request.

## Abbreviations

3D: Three-dimensional
CT: Computed tomography
DDH: Developmental dysplasia of hip
DICOM: Digital imaging and communications in medicine
STL: Standard Tessellation Language
LCP: Locking Compression Plate
